# Optimization of Thyme, Cinnamon, and Black Seed Oil Combinations for Enhanced Antibacterial and Antioxidant Efficacy: Mixture Design and In Silico Insights

**DOI:** 10.3390/ph19030372

**Published:** 2026-02-26

**Authors:** Mahmoud S. Maher, Dina A. Altwiley, Dalal M. Alkuraythi, Mahmoud M. A. Moustafa, Mary S. Khalil, Tarek A. A. Moussa, Nawal Magdy

**Affiliations:** 1 Botany and Microbiology Department, Faculty of Science, Cairo University, Giza 12613, Egypt; 2 Department of Biological Sciences, University of Jeddah, Jeddah 23890, Saudi Arabia; daltwaylee@uj.edu.sa (D.A.A.);; 3 Genetics and Genetic Engineering Department, Faculty of Agriculture, Benha University, Benha 13736, Egypt; mahmoud.mustafa@fagr.bu.edu.eg

**Keywords:** plant-derived oils, synergism, antibacterial, antioxidant, simplex–centroid mixture design, in silico analysis

## Abstract

**Background:** Oils from medicinal plants, including thyme (*Thymus vulgaris*), cinnamon (*Cinnamomum verum*), and black seed (*Nigella sativa*), are recognized for their antibacterial and antioxidant properties. While several studies have investigated individual oils and binary combinations, fewer reports have examined ternary mixtures using systematic optimization approaches. Accordingly, the present study aimed to optimize the antibacterial and antioxidant performance of combinations of these three plant-derived oils using a statistical mixture design strategy supported by in silico exploration. **Methods:** An Augmented Simplex Centroid Mixture Design was applied to evaluate the individual and combined effects of thyme, cinnamon, and black seed oils. Antibacterial activity was assessed by determining the minimum inhibitory concentrations (MICs) against *Escherichia coli* and *Staphylococcus aureus*, while antioxidant activity was measured using the DPPH radical scavenging assay (IC_50_). The experimental data were fitted to a special cubic model, and model validity was verified through ANOVA parameters, including F-values, R^2^, and adjusted R^2^. Multi-response optimization was performed using a desirability function. Potential interactions among oils were further examined using checkerboard assays. Molecular docking and ADMET predictions were conducted as supportive, hypothesis-generating tools. **Results:** The special cubic model was statistically significant for all responses (*p* < 0.0001), with R^2^ values of 0.9763, 0.9944, and 0.9841 for MIC*_E. coli_*, MIC*_S. aureus_*, and DPPH IC_50_, respectively. Response surface analysis and multi-response optimization identified the optimal oil mixture as thyme (41.7%), cinnamon (41.7%), and black seed (16.7%), achieving MIC values of 0.5 µL/mL for *E. coli* and 0.517 µL/mL for *S. aureus*, and a DPPH IC_50_ of 5.32 ± 0.52 mg/mL. Checkerboard assays confirmed synergistic interactions for the optimized formulation, with ΣFIC values of 0.15 and 0.29 against *E. coli* and *S. aureus*, respectively. Cytotoxicity testing of the optimized formulation on WI-38 normal fibroblasts indicated high cell viability (>92%) at all tested concentrations. In silico docking showed favorable binding affinities of major oil constituents with bacterial targets, and ADMET analysis suggested acceptable pharmacokinetic and safety profiles. **Conclusions:** The study demonstrated that specific combinations of thyme, cinnamon, and black seed oils can synergistically enhance antibacterial and antioxidant activities. The optimized formulation exhibited enhanced antibacterial and antioxidant activities with minimal cytotoxicity, while in silico analyses provided supportive mechanistic insights.

## 1. Introduction

In public health, antimicrobial resistance (AMR) is a critical global health challenge that results in millions of deaths annually and imposes significant economic burdens, particularly in developing countries [1]. Misuse of antibiotics without proper medical guidance exacerbates this problem, emphasizing the need for alternative antimicrobial strategies [2,3]. Another challenge in the food industry is the use of synthetic preservatives, such as benzoates, nitrites, butylated hydroxyanisole, polyphosphates, and tocopherols, to inhibit microbial growth and prevent oxidation. However, their high concentrations may pose health risks, including toxicity and carcinogenicity [4]. Along with the growing global demand for eco-friendly, natural bioactive compounds, there has been renewed scientific interest in medicinal and aromatic plants as promising alternatives to synthetic chemicals. These plants are rich sources of diverse secondary metabolites, including phenolics, terpenoids, flavonoids, and alkaloids, which exhibit a wide range of biological activities. Their derivatives are particularly valued for their antimicrobial and antioxidant properties, providing innovative and sustainable strategies for managing bacterial infections caused by both Gram-positive and Gram-negative pathogens. Such natural products have shown remarkable potential in the healthcare, pharmaceutical, and food industries, where the overuse of synthetic chemicals has raised environmental and health concerns [5,6,7,8]. Consequently, there is a growing interest in plant-derived natural compounds such as essential and fixed oils, which contain diverse bioactive molecules generally recognized as safe (GRAS) and are less likely to induce microbial resistance [9,10,11,12]. Plant-derived oils exert their antibacterial activity primarily by disrupting bacterial cell membranes, increasing permeability, causing leakage of intracellular constituents, and inhibiting toxin production [13]. In addition to their antimicrobial activity, oils are well known for their strong antioxidant potential, mainly attributed to the presence of phenolic compounds, which can neutralize free radicals, inhibit lipid peroxidation, and protect cellular structures from oxidative stress [14]. Despite these benefits, their practical application is limited by the need for high concentrations to achieve effective antimicrobial or antioxidant activity [15].

To address these limitations, combining different Plant-derived oils has emerged as an effective strategy. Synergistic interactions between such oils can enhance antimicrobial and antioxidant effects while reducing the required concentrations, minimizing sensory alterations and potential side effects [10,16]. Such synergistic mixtures not only broaden the spectrum of activity and overcome microbial resistance but also reduce costs [17,18]. Previous research has demonstrated that thyme EO, cinnamon EO, and black seed FO individually possess significant antibacterial and antioxidant potential in vitro [19,20,21,22,23]. However, the interactive effects among these three oils have not been investigated. Most earlier studies focused on binary mixtures, neglecting the potentially more complex yet effective ternary combinations that could yield superior bioactivity through multiple synergistic interactions [24,25].

Recent advancements in statistical optimization techniques, such as the simplex–centroid mixture design, have facilitated a more systematic and quantitative evaluation of oil combinations. This approach allows researchers to model the relationship between the mixture proportions and the biological response, thereby identifying the optimal blend composition with minimal experimental effort. When integrated with molecular docking studies, this optimization provides a comprehensive understanding of the molecular mechanisms underlying observed bioactivities by predicting interactions between oil constituents and target bacterial enzymes. Furthermore, evaluating the ADMET (Absorption, Distribution, Metabolism, Excretion, and Toxicity) characteristics of these constituents provides valuable insights into their potential bioavailability, safety, and suitability as therapeutic candidates [26].

Therefore, the present study aims to evaluate the antimicrobial and antioxidant activities of thyme, cinnamon EOs, and black seed FO, both individually and in various binary and ternary combinations, using the simplex–centroid mixture design. Developed mathematical models will help predict the most potent combinations against selected bacterial strains. Additionally, molecular docking and ADMET profiling were performed to investigate the binding affinity, orientation, interaction mechanisms, pharmacokinetic behavior, and safety of the major oil constituents.

## 2. Results

### 2.1. GC-MS Analysis

The GC–MS analysis of thyme oil revealed a complex mixture predominantly composed of oxygenated monoterpenes and monoterpene hydrocarbons. The major constituents were thymol (23.12%), eucalyptol (20.48%), p-cymene (18.70%), estragole (11.12%), and 1-terpineol (10.18%). Other notable compounds included α-terpineol (5.53%), α-terpinene (4.20%), and borneol (2.14%). Minor constituents, including γ-terpinene, linalool, β-pinene, and caryophyllene, were also detected.

The chemical profile of cinnamon oil was strongly dominated by aromatic aldehydes, with (E)-cinnamaldehyde (89.82%) representing the primary constituent. Benzyl alcohol (7.97%) was detected as the second major component, while benzaldehyde (0.59%), benzyl cinnamate (0.47%), trans-cinnamic acid (0.16%), and 14-hydroxycaryophyllene (0.16%) were present in small amounts.

The GC–MS chromatogram of black seed oil displayed five peaks corresponding to major constituents. The predominant compound was 9,12-octadecadienoic acid (Z,Z)- (linoleic acid, 74.38%), followed by n-hexadecanoic acid (palmitic acid, 12.41%), p-cymene (6.73%), and thymoquinone (4.47%). Alpha-thujene (1.22%) was detected as a minor component (Table 1). The GC–MS chromatograms are presented in the Supplementary Materials (Figures S1–S3).

### 2.2. Antibacterial Activity of Oils Compared to Standard Antibiotics

**Figure 1:**
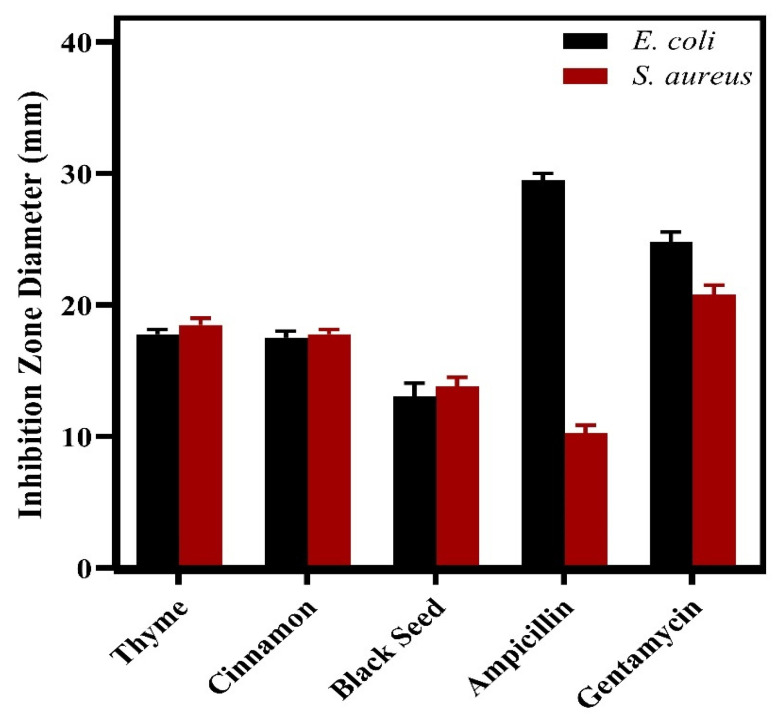
Inhibition zone diameters (IZ, mm) of individual oils and standard antibiotics against *E. coli* and *S. aureus*.

The antibacterial activities of the individual oils were evaluated against *Escherichia coli* and *Staphylococcus aureus*, and the results are presented in Figure 1, showing the inhibition zone diameters (IZ, mm) for each oil compared to the standard antibiotics.

### 2.3. Antioxidant Activity of Individual Oils

**Figure 2:**
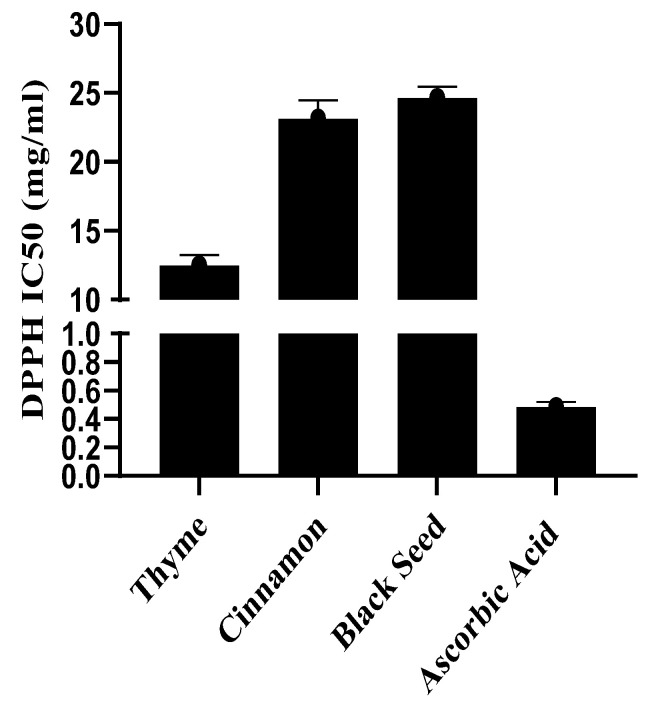
DPPH IC_50_ (mg/mL) of thyme, cinnamon, and black seed oils compared to ascorbic acid.

Thyme oil exhibited the strongest antioxidant activity among the tested oils, with the lowest IC_50_ value, indicating a high free radical-scavenging capacity. In contrast, cinnamon and black seed oils exhibited higher IC_50_ values, indicating moderate antioxidant activity. Ascorbic acid, used as a positive control, showed a markedly lower IC_50_ value than all tested essential oils, confirming its superior antioxidant potency (Figure 2).

### 2.4. Optimization of Oil Combinations Using Experimental Mixture Design

#### 2.4.1. Formulation Design and Interaction Effects on Antibacterial and Antioxidant Responses

Table 2 summarizes the antibacterial and antioxidant activities of thyme, cinnamon, and black seed oils and their binary and ternary combinations, as determined by the Augmented Simplex Centroid Design. The minimum inhibitory concentration (MIC) values against *Escherichia coli* and *Staphylococcus aureus*, along with the DPPH IC_50_ values, revealed notable variations depending on the proportions of the individual oils.

Among the tested blends, the combination of thyme and cinnamon in equal proportions and the ternary mixture containing equal proportions of thyme, cinnamon, and black seed oils exhibited the strongest antibacterial activity, both recording the lowest MIC value of 0.781 μL/mL against *E. coli* and *S. aureus*. Formulations containing higher proportions of thyme or cinnamon oils consistently exhibited stronger antibacterial effects, as evidenced by lower MIC values (0.781–1.56 μL/mL). In contrast, black seed oil, as a single component, showed the weakest antibacterial activity, with the highest MIC value (6.25 μL/mL). Binary and ternary mixtures incorporating black seed oil demonstrated improved antibacterial activity compared to the pure oil, particularly when combined with thyme or cinnamon.

Antioxidant activity, assessed using the DPPH radical scavenging assay, also showed a strong dependence on mixture composition. Several ternary and binary formulations displayed enhanced antioxidant performance compared to single-component systems, with response values ranging from 6.27 ± 0.80 to 15.62 ± 1.27. In contrast, formulations dominated by a single oil generally exhibited higher IC_50_ values, indicating lower antioxidant efficiency.

#### 2.4.2. Statistical Validation of the Model

Statistical analysis of the experimental response data was performed to validate the special cubic mixture models established for each tested response, describing the relationship between the proportions of thyme, cinnamon, and black seed oils and their antibacterial or antioxidant effects. The ANOVA results, summarized in Table 3 and presented in detail in (Tables S1–S3), indicated that all three models were statistically significant (*p* < 0.0001). For MIC against *E. coli*, the model F-value was 89.43 (*p* < 0.0001), while for MIC against S. aureus, the model F-value was 386.00 (*p* < 0.0001), demonstrating the significance of the linear mixture and most interaction terms. Similarly, the model for DPPH IC_50_ was highly significant (F = 134.20, *p* < 0.0001), indicating that the mixture composition strongly influenced antioxidant activity. The coefficients of determination (R^2^) were high for all responses (*E. coli*: 0.9763; *S. aureus*: 0.9944; DPPH: 0.9841), confirming close alignment between the experimental observations and the predicted outcomes of the fitted models (Figure S4). The predicted R^2^ values are in reasonable agreement with the Adjusted R^2^, confirming model reliability and predictive capability. Furthermore, the Adequate Precision values of 29.768, 62.991, and 32.636 exceeded the threshold value of 4, indicating an adequate signal-to-noise ratio and confirming that the models can be effectively used to navigate the experimental design space.

To further quantify the influence of the individual essential oils and their combinations on antibacterial and antioxidant activities, special cubic mixture models were developed. These models describe the relationship between the component fractions (Thyme, Cinnamon, and Black Seed oils) and the observed responses, allowing both prediction and interpretation of the experimental results within the tested design space.

For MIC*_E. coli_*, the regression model in terms of actual component fractions was:

$${\mathrm{MIC}}_{E.\;\mathrm{coli}}=2.93\;A+3.14\;B+6.41\;C-9.72\;AB-6.31\;AC-15.26\;BC+1.65\;ABC$$

where A, B, and C  represent Thyme, Cinnamon, and Black Seed oils, respectively, and the interaction terms (*AB*, *AC*, *BC*, *ABC*) represent the combined effects of the oils. In this model, the ternary interaction (*ABC*) was not statistically significant (*p* = 0.8284), whereas all other terms were significant (*p* ≤ 0.05).

For MIC*_S. aureus_*, all linear, binary, and ternary coefficients were significant, and the model was expressed as:

$${\mathrm{MIC}}_{S.\;\mathrm{aureus}}=1.47\;A+1.61\;B+6.30\;C-3.20\;AB-3.20\;AC-9.17\;BC-15.70\;ABC$$

Similarly, for antioxidant activity (DPPH IC_50_), all coefficients were significant, and the model was:

$${\mathrm{DPPH}\;\mathrm{IC}}_{50}=12.93\;A+23.31\;B+24.24\;C-32.88\;AB-26.49\;AC+15.75\;BC-246.26\;ABC$$

These actual component models allow the prediction of responses for given levels of each essential oil within the tested design space. Usually, a negative sign of a coefficient in the fitted model indicates the ability of its factor to decrease the response, whereas a positive sign indicates the ability of a factor to increase the response variable. However, since the goal of this study was to enhance the antibacterial and antioxidant effects, i.e., to minimize the response variables (MIC and IC_50_ values), a negative coefficient reveals the ability of its associated factor or interaction to enhance the biological activity of the oil mixture, whereas a positive coefficient indicates a tendency to weaken the effect or increase the response value.

Because MIC values are determined using two-fold serial dilutions, they represent discrete endpoints rather than continuous measurements. Consequently, the zero pure error observed in the ANOVA reflects measurement resolution constraints rather than the absence of biological variability. Model statistics such as R^2^ and F-values should therefore be interpreted as indicators of goodness-of-fit within the experimental design space, rather than as evidence of deterministic biological behavior.

#### 2.4.3. Response Surface Analysis

**Figure 3:**
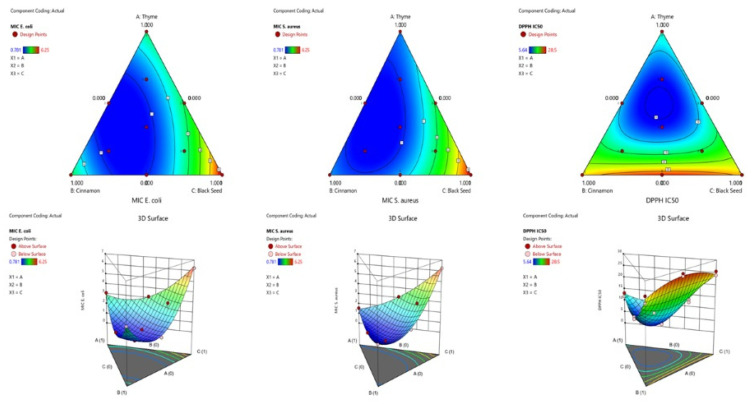
Contour and three-dimensional response surface plots showing the interaction effects of thyme, cinnamon, and black seed oils on each experimental response.

The contour plot and the 3D surface graph, presented as 2D and 3D mixture plots in Figure 3, illustrate the optimal combination of the three oils for maximizing antibacterial activity against *E. coli* and *S. aureus*, as well as antioxidant activity measured by DPPH IC_50_. These visual tools depict the relationship between the biological responses and the concentrations of each oil. The plots employ iso-response curves, which are effective for identifying the precise formulation conditions needed to achieve the most favorable antibacterial and antioxidant outcomes. In the visualizations, the color gradients indicate varying levels of activity: blue regions correspond to the lowest MIC and IC_50_ values, representing the highest antibacterial and antioxidant efficacy, whereas areas shaded from yellow to dark red reflect progressively higher MIC and IC_50_ values, indicating reduced effectiveness.

#### 2.4.4. Integrated Multi-Response Optimization

**Figure 4:**
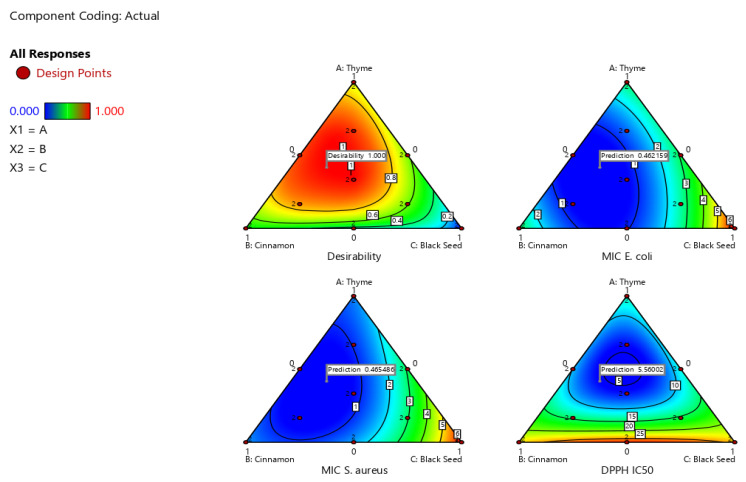
Desirability plot showing the optimal mixture with predicted responses. The top-left plot shows the overall desirability function for optimizing responses. The top-right and bottom-left plots show predicted MIC values against *E. coli* and *S. aureus*, respectively, while the bottom-right plot displays predicted DPPH IC_50_ values.

The integrated multi-response desirability tool was applied to determine the optimal combination of cinnamon, thyme, and black seed oils to maximize antibacterial activity against *E. coli* and *S. aureus*, as well as antioxidant activity, measured as DPPH IC_50_. Desirability values range from 0 to 1, where 0 represents an unacceptable response and 1 indicates the maximum desired effect. (Figure 4) depicts the optimal mixture, consisting of 41.7% thyme, 41.7% cinnamon, and 16.7% black seed, achieving the lowest MIC and IC_50_ values and a desirability of 100.

#### 2.4.5. Experimental Verification of the Assumed Model

To confirm the validity of the proposed special cubic mixture model, experimental validation was performed using the optimized oil formulation predicted by the desirability function. This analysis is crucial for verifying the accuracy of these models in predicting antibacterial activity against *E. coli* and *S. aureus*, as well as antioxidant activity measured by DPPH IC_50_. The model’s reliability is supported by the close alignment between experimental results and predicted values, as shown in Table 4, which demonstrates a strong correlation and confirms its effectiveness in practical applications.

### 2.5. Synergistic Effect Evaluation of the Developed Formulations by Checkerboard Assay

The synergistic antibacterial activity of the developed formulations was further evaluated using a checkerboard assay to confirm interactions among the oils. The data for the binary, ternary, and optimal mixtures are presented in Table 5. Several oil combinations displayed pronounced synergistic effects against both *E. coli* and *S. aureus*. Among all tested formulations, the ternary mixture containing equal proportions of thyme, cinnamon, and black seed oils (0.333:0.333:0.333) exhibited strong synergism, with ΣFIC values of 0.208 for *E. coli* and 0.375 for *S. aureus*. Similarly, the binary mixture of thyme and cinnamon at a 1:1 ratio demonstrated significant synergy (ΣFIC = 0.25). Notably, the statistically optimized mixture, containing thyme (0.417), cinnamon (0.417), and black seed (0.167), exhibited the strongest synergistic effect, with ΣFIC values of 0.15 and 0.29 against *E. coli* and *S. aureus*, respectively, confirming the enhanced potency of the optimized formulation.

For combinations yielding ΣFIC values near interpretative thresholds, synergistic interactions should be considered suggestive rather than absolute and interpreted in the context of biological variability and assay resolution.

### 2.6. Cytotoxicity Evaluation Against the Normal Cell Line (WI-38)

**Figure 5:**
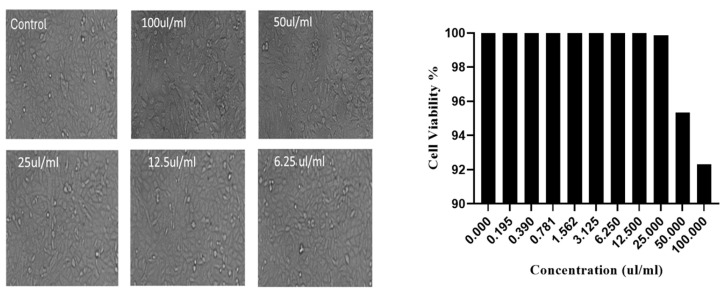
Cytotoxicity of the optimized oil formulation against normal WI-38 cells after 48 h of exposure. Concentrations are expressed in µL/mL.

The cytotoxic effect of the optimized formulation on normal WI-38 cells was assessed by measuring cell viability and cytotoxicity after 48 h of exposure at concentrations ranging from 0.195 to 100 µL/mL (Figure 5). Across all tested concentrations, WI-38 cells exhibited high viability, ranging from 92.3% to 100%, with no statistically significant difference compared to the untreated control. Correspondingly, cytotoxicity percentages remained very low, not exceeding 7.67%, even at the highest concentration tested (100 µL/mL).

### 2.7. Docking Analysis

#### 2.7.1. Docking Interpretations—Cinnamaldehyde, Linoleic Acid, and Thymol Pose Across Targets

**Figure 6:**
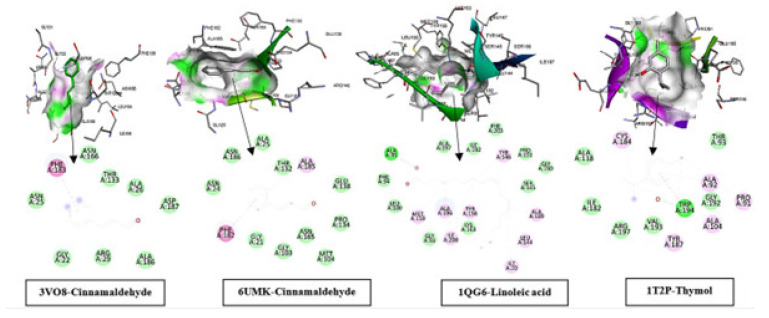
Compares ligand chemotypes across targets. Cinnamaldehyde (3VO8, 6UMK) is a compact phenylpropanoid with an aromatic ring and α, β-unsaturated aldehyde, enabling π-driven recognition (stacking) while remaining largely hydrophobic. Linoleic acid (1QG6) is a flexible C18 polyunsaturated fatty acid that inserts deeply into a lipophilic channel, anchored mainly by its polar carboxylate. Thymol (1T2P) is a lipophilic phenolic monoterpene, stabilized by hydrophobic packing and one H-bond donor. The arrows show the pose of ligand-protein interactions from 3D to 2D.

Molecular docking was used as an exploratory, hypothesis-generating approach to examine whether major constituents might adopt plausible binding poses within selected bacterial targets (Figure 6). For cinnamaldehyde, linoleic acid, and thymol, the predicted poses were predominantly stabilized by hydrophobic and π-type interactions within non-polar pockets located near substrate- or nucleotide-associated regions of the target proteins. Importantly, docking poses and qualitative interaction maps do not establish binding affinity or mechanism; no experimental target engagement assays, pose validation, or molecular-dynamics refinement were performed. Accordingly, the docking results are discussed as supportive, computational hypotheses rather than definitive mechanistic proof.

The binding poses and 2D interaction fingerprints of selected ligands against key bacterial targets were visualized, including FtsZ (cell-division GTPase) from *Staphylococcus aureus* (PDB: 3VO8) and *Escherichia coli* (PDB: 6UMK) complexed with cinnamaldehyde, FabI (enoyl-ACP reductase) from *E. coli* (PDB: 1QG6) complexed with linoleic acid, and Sortase A from *S. aureus* (PDB: 1T2P) complexed with thymol; in each panel, the upper view showed the ligand positioned within the binding pocket with surrounding residues rendered as sticks and a semi-transparent surface highlighting pocket shape complementarity, whereas the lower view summarized pocket-lining residues and non-covalent contacts in the Discovery Studio 2D interaction map. In the Discovery Studio 2D interaction maps, 3VO8–cinnamaldehyde showed π–π stacking with PHE183 and van der Waals contacts with ASN166, THR133, ALA26, ASP187, ALA186, ARG29, GLY22, and ASN25; 6UMK–cinnamaldehyde showed π–π stacking with PHE182, a π–alkyl interaction with ALA185, and van der Waals contacts with ALA25, ASN186, THR132, GLU138, PRO134, ASN165, MET104, GLY103, GLY21, and ASN24; 1QG6–linoleic acid formed a conventional hydrogen bond with ALA95, alkyl/π–alkyl interactions with MET159, ALA196, ILE200, TYR156, TYR146, ALA189, LEU144, and ILE20, and van der Waals contacts with PHE94, LEU100, GLY93, LYS163, ALA197, ILE192, PRO191, GLY190, SER145, and PHE203; and 1T2P–thymol formed a conventional hydrogen bond with TRP194, a π–π T-shaped interaction with TYR187, alkyl/π–alkyl interactions with CYS184, ALA92, PRO91, and ALA104, and van der Waals contacts with ALA118, ILE182, ARG197, VAL193, GLY192, and THR93 as shown in (Figure 6).

#### 2.7.2. Docking Interpretations—Eucalyptol Across Targets

**Figure 7:**
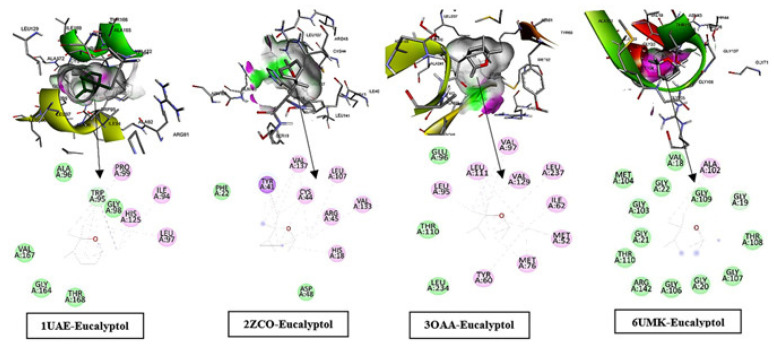
Shows eucalyptol docked into the binding pockets of four proteins (1UAE, 2ZCO, 3OAA, 6UMK), with a 3D pose and a 2D interaction fingerprint. Across complexes, recognition is mainly hydrophobic: extensive van der Waals contacts dominate, supported by alkyl/π–alkyl interactions with nearby residues. Each protein displays a distinct set of contacting amino acids, highlighting similar interaction types but pocket-specific residue contributors in four docking models. The arrows show the pose of ligand-protein interactions from 3D to 2D.

Additional docking simulations for eucalyptol that can occupy hydrophobic cavities in the investigated proteins (Figure 7), with polar functional groups occasionally forming hydrogen-bond or electrostatic contacts to nearby residues. The black seed oil marker (linoleic acid) poses were generally consistent with accommodation of a long aliphatic chain within lipid-like tunnels. As above, these in silico poses are qualitative and do not quantify binding under physiological conditions; mechanistic interpretations should therefore remain cautious until supported by biochemical inhibition and/or binding studies.

Eucalyptol ligand was mapped within the binding sites of MurA (PDB: 1UAE; relevant to both *E. coli* and *S. aureus*), FtsZ (PDB: 2ZCO and 6UMK; relevant to both *E. coli* and *S. aureus*), and F1F0-ATP synthase/ATPase (PDB: 3OAA; *E. coli*), and the 2D interaction fingerprints indicated that binding was driven mainly by nonpolar contacts with limited directional contributions. In MurA (1UAE), eucalyptol was stabilized by van der Waals contacts (ALA96, TRP95, GLY98, VAL167, GLY164, THR168) together with alkyl/π–alkyl interactions (PRO99, ILE94, HIS125, LEU97) and additional weak contact features (as annotated in the DS legend). In FtsZ (2ZCO), the ligand was surrounded by van der Waals residues (PHE22, ASP48) and reinforced by π-type/hydrophobic interactions, including π-sigma with TYR41 and alkyl/π–alkyl contacts with VAL137, LEU107, VAL133, CYS44, ARG45, and HIS18. In F1F0-ATP synthase/ATPase (3OAA), eucalyptol was accommodated primarily through alkyl/π–alkyl interactions with LEU95, LEU111, VAL97, VAL129, LEU237, ILE62, MET52, MET76, and TYR60, alongside van der Waals contributions from GLU96, THR110, and LEU234. In FtsZ (6UMK), binding was dominated by an extensive van der Waals network (MET104, VAL18, GLY22, GLY109, GLY19, THR108, GLY107, GLY20, GLY106, ARG142, THR110, GLY21, GLY103) with an additional alkyl contact (ALA102), consistent overall with predominantly hydrophobic stabilization of eucalyptol across these targets as shown in (Figure 7).

#### 2.7.3. Docking Interpretations *p*-Cymene Across Targets

**Figure 8:**
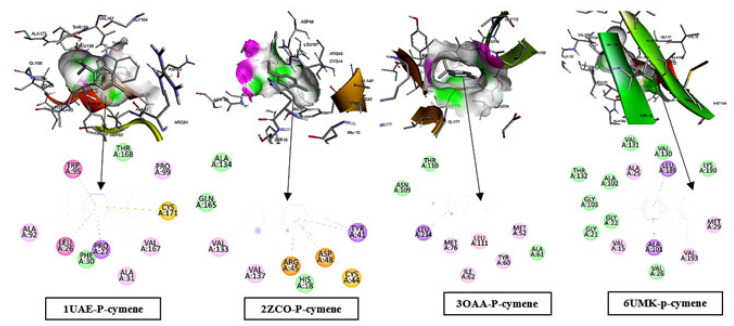
Depicts p-cymene docked in the binding pockets of four proteins (1UAE, 2ZCO, 3OAA, and 6UMK), showing 3D poses and 2D interaction maps. Binding is largely lipophilic, dominated by van der Waals contacts with additional alkyl/π–alkyl interactions and only minor π-type contributions. Each complex involves a characteristic set of hydrophobic residues surrounding the ligand, with occasional π contacts (e.g., TYR41 in 2ZCO and LEU234 in 3OAA). The arrows show the pose of ligand-protein interactions from 3D to 2D.

The 2D interaction maps showed that p-cymene bound predominantly through hydrophobic contacts across all targets, with additional π-type interactions depending on the pocket environment: in MurA (1UAE–p-cymene) the ligand was stabilized mainly by alkyl/π–alkyl contacts with ALA92, LEU26, ALA31, VAL167, and PRO99, together with π–π stacking with TRP95, a π–sigma contact with PRO27, a π–sulfur interaction with CYS171, and van der Waals contributions from THR168 and PHE30; in FtsZ (2ZCO–p-cymene) binding involved alkyl/π–alkyl interactions with VAL133 and VAL137, a π–sigma interaction with TYR41, π–cation/π–anion features with ARG45 and ASP48, a π–sulfur contact with CYS44, and van der Waals contacts with HIS18, GLN165, and ALA134; in F1F0-ATP synthase/ATPase (3OAA–p-cymene) the ligand was supported by alkyl/π–alkyl contacts with LEU111, MET76, MET52, TYR60, and ILE62, with a π–sigma interaction involving LEU234 and van der Waals contributions from THR110, ASN109, and ALA61; and in FtsZ (6UMK–p-cymene) the interaction pattern was dominated by van der Waals contacts with THR132, ALA102, GLY103, GLY22, GLY21, VAL26, VAL131, VAL130, and LYS190, complemented by alkyl/π-type stabilization from ALA25, VAL15, MET29, and VAL193 and π–sigma contacts with ALA101 and LEU189, collectively indicating that p-cymene binding was largely lipophilic with target-specific π-interaction reinforcement (Figure 8).

### 2.8. Drug-likeness and ADME-Toxicity

Predicted physicochemical properties (Table 6) were used only for preliminary drug-likeness screening of five major constituents. Most compounds fell within common ranges for molecular weight and hydrogen-bonding capacity, while linoleic acid exhibited high lipophilicity (high predicted logP), which may limit aqueous solubility and oral absorption unless formulated appropriately. These in silico estimates do not replace experimental solubility, permeability, or bioavailability measurements.

ADME/toxicity outputs (Table 7) were interpreted as computational screening results. Predicted intestinal absorption, mutagenicity (AMES), hepatotoxicity, skin sensitization, BBB/CNS penetration, and CYP-related interaction flags vary by compound and are model-dependent; therefore, they should not be used to claim safety or therapeutic applicability. The predictions instead help prioritize compounds for follow-up experimental evaluation (e.g., cytotoxicity, metabolic stability, and in vivo tolerability). Predictions were generated using commonly used web tools such as SwissADME (swissadme.ch) and pkCSM (biosig.lab.uq.edu.au/pkcsm/).

**Figure 9:**
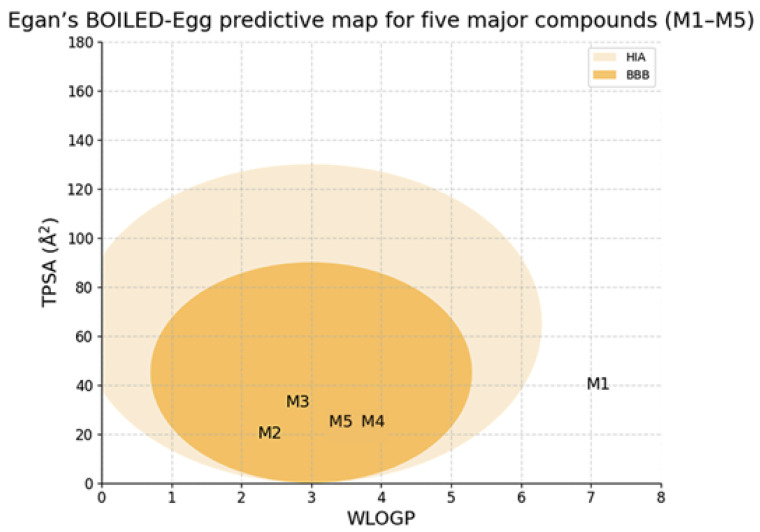
Application of the BOILED-Egg model (SwissADME) to five major compounds (M1–M5).

The BOILED-Egg model (SwissADME; WLOGP vs. TPSA) was additionally used to visualize predicted passive gastrointestinal absorption and BBB permeation tendencies (Figure 9). As with other ADME tools, these outputs are approximate and should be considered alongside experimental pharmacokinetic and toxicity studies.

## 3. Discussion

The present study successfully optimized the antibacterial and antioxidant potentials of oil combinations using a mixture design strategy integrated with statistical and in silico analyses. The findings highlight synergistic interactions among thyme (*Thymus vulgaris*), cinnamon (*Cinnamomum verum*), and black seed (*Nigella sativa*) oils, which, when used collectively, enhance biological efficacy compared to their individual applications.

The antibacterial evaluation revealed that both *E. coli* and *S. aureus* were susceptible to the tested essential oils. Gram-negative *E. coli* displayed relatively lower susceptibility to the individual oils, which can be attributed to the protective outer membrane, which contains hydrophilic lipopolysaccharides (LPS) that limit the penetration of oil components [27]. Among all formulations, the binary mixture of thyme and cinnamon and the ternary mixture containing equal proportions of the three oils demonstrated the strongest antibacterial activities, with the lowest MIC values. These results clearly indicate a synergistic interaction between the oils, particularly between thyme and cinnamon.

The enhanced antibacterial activity of such mixtures may be attributed to the combined action of their major bioactive constituents—thymol, cinnamaldehyde, linoleic acid, and thymoquinone. These compounds act synergistically by disrupting bacterial cell membranes, increasing permeability, interfering with key metabolic enzymes, and causing leakage of intracellular contents, thereby amplifying the overall antibacterial effect compared to the individual oils [2,15,17,28]. Previous studies have reported similar synergistic effects between phenolic-rich oils, in which interactions between hydroxylated monoterpenes and aldehyde constituents enhanced bacterial inhibition [29]. Several studies have demonstrated the synergistic antibacterial effects of various oil combinations, underscoring their potential as natural antimicrobial systems. For example, a previous investigation reported that a ternary blend of *Moringa oleifera*, *Cinnamomum verum*, and *Nigella sativa* oils exhibited strong synergistic effects against *Staphylococcus aureus*, significantly lowering the MIC values compared to individual oils [6]. Similarly, another study demonstrated that combining *Origanum vulgare* (oregano), *Thymus vulgaris* (thyme), and *Cymbopogon citratus* (lemongrass) oils via a simplex–centroid mixture design yielded optimized blends that simultaneously inhibited *Salmonella enterica*, *Escherichia coli*, and *Staphylococcus aureus* [10].

Unlike MIC values, which are discrete endpoints constrained by two-fold dilution steps, DPPH scavenging activity represents a continuous response, allowing finer resolution of variance and model residuals. These intrinsic differences were taken into account when interpreting model performance and statistical outputs. The DPPH assay demonstrated a pronounced synergistic effect in antioxidant potential among the oil combinations. The ternary blend containing equal proportions of thyme, cinnamon, and black seed oils exhibited the greatest radical-scavenging capacity, surpassing the activity of the individual oils. Similar findings have previously been reported, demonstrating that enriching oils with polyphenolic mixtures or plant extracts significantly enhanced their DPPH activity through cooperative antioxidant mechanisms [30,31].

The statistical validation confirmed the robustness of the developed cubic mixture models for all tested responses. The high R^2^ and adjusted R^2^ values, along with the non-significant lack-of-fit results, demonstrate that the models accurately describe the relationships between oil proportions and biological activities. The agreement between experimental and predicted MIC and IC_50_ values further supports the predictive strength and practical applicability of the models [13]. The integrated desirability function identified several optimal blends with a desirability index of 1.0, and one was selected for model validation. This optimized mixture achieved the lowest MIC and IC_50_ values, confirming the effectiveness of the mixture design in maximizing multi-response optimization. These results, along with other previous studies, underscore the potential of employing simplex–centroid designs in the formulation of natural antimicrobial and antioxidant products [16,32].

The cytotoxicity assessment of the optimized essential oil formulation on WI-38 normal fibroblast cells showed no significant difference compared to the control, with cells remaining viable and exhibiting minimal to negligible cytotoxicity. These results indicate that the tested essential oil does not induce significant cellular damage or inhibition in normal fibroblasts.

The chemical composition of plant-derived oils can vary due to factors such as plant origin, harvest period, and extraction method. This inherent variability may influence biological activity and the reproducibility of results. Nevertheless, slight variations in composition could still contribute to differences in activity, and this should be considered when interpreting the results and comparing them with other studies.

The in-silico docking results were used as exploratory, hypothesis-generating evidence to complement the mixture-design bioassays. Across the five major constituents, predicted poses were dominated by lipophilic complementarity—van der Waals and alkyl/π–alkyl contacts—with limited directional interactions. Cinnamaldehyde adopted plausible poses in FtsZ pockets from *S. aureus* (3VO8) and *E. coli* (6UMK), consistent with prior reports that cinnamaldehyde and related scaffolds can perturb FtsZ function and cell division [33,34,35]. Linoleic acid displayed a tail-in-channel pose in FabI (1QG6), with its carboxylate positioned for anchoring at the channel entrance, which is coherent with fatty-acid accommodation near FASII targets and exogenous fatty-acid utilization contexts [36]. Thymol fits a hydrophobic groove of Sortase A (1T2P) with one potential H-bond contribution, aligning with common anti-virulence SrtA inhibitor binding motifs [37,38,39].

For the weaker polar monoterpenes, eucalyptol and p-cymene were predicted to occupy non-polar cavities in MurA (1UAE), FtsZ (2ZCO/6UMK), and the F1F0/ATP synthase/ATPase target (3OAA) primarily via dispersion forces (Figure 8 and Figure 9). These contact patterns suggest a nonspecific lipophilic association rather than a strongly directed binding mode and should therefore be interpreted cautiously until supported by target-based inhibition, binding, or cellular mechanism assays [40,41,42].

Four compounds met common Lipinski-type thresholds (MW < 500; HBD < 5; HBA < 10) and showed moderate lipophilicity (predicted logP ≈ 2.2–3.3), supporting passive permeability expectations, whereas linoleic acid exhibited high predicted lipophilicity (logP ≈ 7.0), which can reduce aqueous solubility and make oral exposure more formulation-dependent [43,44,45]. Molar refractivity values fell within the typical 40–130 window, consistent with broadly acceptable physicochemical space for small molecules [46,47].

**Figure 10:**
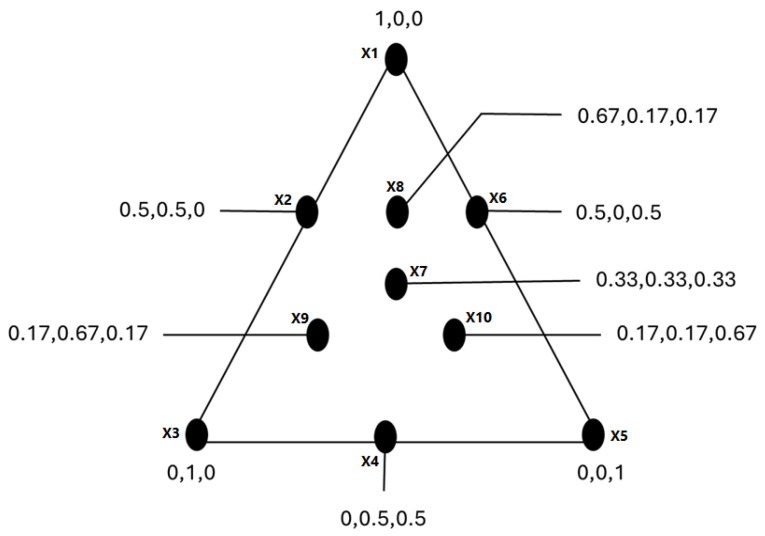
An overview of the simplex–centroid design.

The ADME/toxicity screening trends for the same five constituents should be interpreted as model-dependent prioritization rather than definitive safety/efficacy evidence [48,49,50]. All five showed high predicted intestinal absorption, while BBB/CNS indices suggested greater brain-entry potential for the compact monoterpenes (eucalyptol and p-cymene) than for linoleic acid. CYP interaction flags were limited overall; linoleic acid was predicted as a CYP3A4 substrate and a CYP2C9 inhibitor, whereas the other compounds showed no CYP substrate/inhibitor alerts in this workflow. Skin-sensitization alerts were predicted for cinnamaldehyde and p-cymene, indicating a need for careful irritation/sensitization testing. The BOILED-Egg visualization (Figure 10) provided a qualitative cross-check of passive absorption/BBB tendencies and should be considered alongside experimental pharmacokinetics and toxicity studies [43,44,45].

Despite the promising antibacterial and antioxidant outcomes, the present study has some limitations that should be acknowledged. First, the number of microbial strains tested was limited to *E. coli* and *S. aureus*, which may not fully represent the broad-spectrum activity of the essential oil combinations. Second, only a single antioxidant assay (DPPH) was employed, and other methods, such as ABTS or FRAP, could provide a more comprehensive evaluation of antioxidant potential. Third, the in-silico docking and ADMET predictions, while informative, are exploratory and cannot substitute for experimental confirmation of molecular mechanisms or in vivo pharmacokinetics. Future research should expand the range of microbial targets, include multiple antioxidant assays, and perform in vivo studies to validate safety and efficacy. Additionally, the biological relevance of the mixture design-derived optimal ratios should be further investigated under realistic application conditions, such as therapeutic formulations, to ensure their practical applicability.

## 4. Methodology

### 4.1. Plant-Derived Oils

The oils of thyme (*Thymus vulgaris*), cinnamon (*Cinnamomum verum*), and black seed (*Nigella sativa*) were purchased from the National Research Center (NRC) in Cairo, Egypt. Cinnamon essential oil was obtained from the dried inner bark of *Cinnamomum* verum sourced from Sri Lanka. Thyme essential oil was extracted from the dried leaves of *Thymus vulgaris* (thyme) of the thymol chemotype, cultivated in Upper Egypt from Beni Suef Governorate. Black seed oil was cold-pressed from Nigella sativa (black cumin) seeds originating from Beni Suef Governorate, Egypt. Both cinnamon and thyme oils were produced by steam distillation using a standard Clevenger-type apparatus for 3 h. Black seed oil was cold-pressed at room temperature (25–30 °C). All oils were stored in airtight amber glass vials at 4 °C, protected from light, until use [51,52,53].

### 4.2. Analysis of the Chemical Composition of Oils by GC-MS Spectroscopy

The sample was dissolved in dichloromethane and then injected. The GC-MS system (Agilent Technologies, Santa Clara, CA, USA) was equipped with a gas chromatograph (7890B) and a mass spectrometer (5977A) at the Central Laboratories Network of the National Research Centre, Cairo, Egypt. The GC was equipped with an HP-5MS column (15 m × 0.25 mm internal diameter, 0.25 μm film thickness). Analyses were carried out using Hydrogen as the carrier gas at a flow rate of 1.1 mL/min at a splitless injection volume of 1.0 µL and the following temperature program: 40 °C for 1 min; rising at 10 °C/min to 200 °C and held for 1 min; rising at 20 °C/min to 220 °C and held for 1 min; rising at 30 °C/min to 300 °C and held for 3 min. The injector and detector were held at 250 °C and 300 °C, respectively. Mass spectra were obtained by electron ionization (EI) at 70 eV, using a spectral range of *m*/*z* 33–600 and a solvent delay of 1.60 min. The mass temperature was 230 °C, and the Quad was 150 °C. Identification of the different constituents was determined by comparing the fragmentation patterns of the spectra with those stored in the Wiley and NIST Mass Spectral Libraries and by comparing calculated retention indices with those reported in the literature [54,55].

### 4.3. Preparation of Oil Emulsions

To facilitate the incorporation of oils, they were emulsified in 2% dimethyl sulfoxide (DMSO) by mixing 50 μL of each oil or oil combination with 950 μL of 2% DMSO [56]. A preliminary assessment confirmed that 2% DMSO exhibited no inhibitory effect on the growth of the tested bacterial strains.

### 4.4. Antibacterial Assays

#### 4.4.1. Bacterial Strains

Two standard bacterial strains were used to evaluate the antibacterial activity of the oils, either individually or in combination: *Escherichia coli* ATCC 25922 and *Staphylococcus aureus* ATCC 29213. These strains were obtained from the American Type Culture Collection (ATCC) and selected as representative models of Gram-negative and Gram-positive bacteria, respectively. Both strains were maintained on Mueller–Hinton agar (MHA) slants at 4 °C.

#### 4.4.2. Disc Diffusion Experiments

The antibacterial activity of the oils was tested using the agar disc diffusion method in accordance with the recommendations of the Clinical and Laboratory Standards Institute (CLSI) [57]. Specifically, Mueller–Hinton (MH) agar plates were prepared and uniformly inoculated with the respective bacterial strains using a sterile swab, with the bacterial suspension adjusted to 0.5 McFarland standard. Sterile paper discs were impregnated with the oil. The plates were incubated at 37 °C for 24 h, after which the zones of inhibition around each disc were measured in millimeters. Both gentamicin and ampicillin were used as positive controls. All experiments were carried out in independent triplicates to ensure accuracy and reproducibility.

#### 4.4.3. MIC Determination

The minimum inhibitory concentration (MIC) was determined by broth microdilution in sterile 96-well microtiter plates. MH broth was used as the diluent, and the bacterial suspension turbidity was adjusted to 0.5 McFarland standard. Twofold serial dilutions of each oil, alone or in combination, were prepared in the wells, with a final volume of 100 μL per well. The oil concentrations ranged from 25 μL/mL to 0.0244 μL/mL. The plates were incubated at 37 °C for 24 h. Following incubation, resazurin (0.015%) was added as a redox indicator to assess bacterial viability. and the MIC was defined as the lowest concentration of the oil that prevented resazurin from changing from blue to pink, indicating inhibition of bacterial growth [58,59].

#### 4.4.4. Checkboard Assay

The interactions among thyme, cinnamon, and black seed oils against the selected bacterial strains were evaluated using the checkerboard approach as previously described [60,61]. This assay was conducted to determine the type of interaction (synergistic, additive, or antagonistic) between the oils in both binary and ternary combinations generated through the simplex–centroid mixture design. The interaction was quantified as the fractional inhibitory concentration (FIC) index, calculated by summing the FIC values of the individual oils in each combination. The FIC value of each oil was defined as the ratio between the MIC of the oil in combination and its MIC when tested individually, according to the following equations:

$${\mathrm{FIC}}_{\mathrm{oil}}=\frac{\mathrm{MIC}\;(\mathrm{oil}\;\mathrm{in}\;\mathrm{combination})}{\mathrm{MIC}\;(\mathrm{oil}\;\mathrm{alone})}$$

$${\mathrm{FIC}}_{\mathrm{index}}={\mathrm{FIC}}_{A}+{\mathrm{FIC}}_{B}(+{\mathrm{FIC}}_{C},\;\mathrm{for}\;\mathrm{ternary}\;\mathrm{mixtures})$$

The obtained FIC index values were interpreted as follows: synergistic effect (S) if FIC ≤ 0.5; additive (AD) if 0.5 < FIC ≤ 1; no interaction (NI) or indifferent if 1 < FIC ≤ 4; and antagonistic effect (AG) if FIC > 4.

### 4.5. Antioxidant Activity Evaluation Using DPPH Radical Scavenging Assay

The antioxidant activity of the three oils and their various combinations, generated using the mixture design, was evaluated using the 2,2-diphenyl-1-picrylhydrazyl (DPPH) radical scavenging assay. This method measures the ability of antioxidants in the samples to quench the stable DPPH free radical, which exhibits a characteristic color change from purple to yellow upon reduction. The procedure was carried out following a modified method described by [62]. Briefly, 50 μL of the oil sample at different concentrations (50, 40, 30, 20, 10, 5, 2.5, 1.25, and 0.78 mg/mL) was mixed with 950 μL of a 0.1 mM DPPH methanolic solution. The reaction mixture was incubated in the dark at room temperature for 30 min to ensure complete reaction. Ascorbic acid was used as a positive control. The absorbance was then recorded at 517 nm using a spectrophotometer. The radical scavenging activity (RSA) was calculated according to the following equation:

$$\mathrm{RSA}\;(\%)=(\frac{A_{b}-A_{x}}{A_{b}})\times 100$$

where $A_{b}$ is the absorbance of the blank (DPPH solution without sample), and $A_{x}$ is the absorbance of the sample. Then the IC_50_ value (the concentration required to scavenge 50% of DPPH radicals) was subsequently determined.

### 4.6. Development of Formulations Using Mixture Design and Mathematical Modeling

#### 4.6.1. Mixture Design

To evaluate the combined antibacterial and antioxidant effects of the selected oil mixtures, a simplex–centroid mixture design based on Scheffé regression models was employed. The centroid design allows systematic investigation of single components, binary mixtures, and ternary combinations while minimizing the number of experimental runs. Three oils were investigated in this design: thyme, cinnamon, and black seed. The independent variables represented the fractional proportions of each oil in the mixture, ranging from 0 to 1, with no constraints. The experimental matrix consisted of single, binary, and ternary combinations of oils, thereby covering the entire mixture space (Figure 10). The vertices of the triangular plot corresponded to the pure components, the midpoints of the edges represented binary mixtures, and the centroid represented the ternary mixture. To improve the estimation of experimental error and enhance model reliability, the entire experimental design was duplicated. The measured responses included the minimum inhibitory concentration (MIC) against *Escherichia coli* and *Staphylococcus aureus*, and the half-maximal inhibitory concentration (IC_50_) for DPPH radical scavenging activity. In a preliminary step, linear, quadratic, cubic, and special cubic least-squares regression models were evaluated using analysis of variance (ANOVA) to identify the most appropriate model for each response. The special cubic model was selected as the most suitable to describe the relationship between mixture composition and all investigated responses.

The special cubic model used for response prediction is expressed as:

Y = α_1_X_1_ + α_2_X_2_ + α_3_X_3_ + α_12_X_1_X_2_ + α_13_X_1_X_3_ + α_23_X_2_X_3_ + α_123_X_1_X_2_X_3_ + ε

where Y is the response (MIC, µL/mL, or IC_50_ in mg/mL), X_1_, X_2_, and X_3_ are the proportions of thyme, cinnamon, and black seed oils, respectively; α_1_, α_2_, and α_3_ represent the linear coefficients; α_12_, α_13_, and α_23_ denote the binary interaction coefficients; α_123_ represents the ternary interaction coefficient; and ε is the residual error [10,26].

#### 4.6.2. Statistical Analysis and Optimization Tools

All experiments, including MIC and DPPH IC_50_ determinations, were performed in triplicate, and the results were expressed as mean values ± standard deviation. Statistical analysis and model fitting were conducted using *Design-Expert.* The parameters obtained from the analysis of variance (ANOVA) included the sum of squares (SS), mean square (MS), degrees of freedom (df), F-values, *p*-values, and the coefficient of determination (R^2^). The statistical significance of the models and individual terms was evaluated at the 5% significance level (α = 0.05). A high F-value and a low *p*-value (*p* < 0.05) indicated that the model terms were significant and that the variability in the data was well explained by the fitted model. The adequacy of the cubic regression model was further verified by evaluating R^2^, adjusted R^2^, and predicted R^2^ values, which measure the degree of correlation between the experimental and predicted responses. Additionally, the lack-of-fit test was used to ensure that the model adequately described the experimental data without significant deviation. For optimization, contour and 3D surface plots were used to visualize component interactions and identify the most effective mixtures. The desirability function helped determine the optimal formulation by balancing the responses to achieve the best overall results. In this function, values range from 0 (undesirable) to 1 (highly desirable), ensuring a practical and efficient optimization process [13].

### 4.7. Assessment of Cytotoxicity of Optimized Mixture Against Normal WI-38 Cell Line

The cytotoxicity of the optimized formulation was evaluated using the MTT assay on WI-38 normal fibroblast cells. Initially, to develop a complete monolayer sheet, the 96-well tissue culture plate was inoculated with 1 × 10^5^ cells/mL (100 µL/well) and incubated at 37 °C for 24 h. After a confluent sheet of cells had been formed, the Growth medium was decanted from the 96-well microtiter plates, and the cells were washed twice. Then they were treated with a tested sample; two-fold dilutions of the tested sample (0.195–100 µL/mL) were prepared in RPMI 1640 medium with 2% serum (maintenance medium). Maintenance medium only was used as a control. After cells were incubated for 48 h at 37 °C, 20 µL of MTT solution (5 mg/mL in PBS) was added to each well. The plate was incubated (37 °C, 5% CO_2_) for 4 h, then it was discarded. The MTT metabolic product (formazan) was resuspended in 200 µL DMSO, then shaken at 150 rpm for 5 min to thoroughly mix the formazan into the solvent. Colorimetric analysis was measured at 560 nm, with background subtraction at 620 nm [63].

### 4.8. In Silico Study

#### 4.8.1. Protein/Ligand Preparation, Docking, and Visualization

Protein structures from the RCSB PDB were cleaned by removing heteroatoms and remote waters, rebuilding missing side chains, and adding hydrogens at pH 7.4. Prepared receptors were saved as PDB files. Ligands were sketched in 2D, converted to 3D, minimized using a small-molecule force field, and exported as PDB and SDF files, preserving pH-appropriate tautomer. Docking was performed using HDOCK (default, whole-receptor search), ranked poses/scores were retrieved, and the top ten were downloaded. Poses were filtered for plausible placement in the FPP/active-site channel, π stacking/hydrophobic interactions, and a hydrogen bond from the phenolic OH; clashes, strained torsions, or fully buried OH groups were rejected. The best-scoring pose was selected for the figures. Figures were created in Chimera and Discovery Studio, with pocket renderings and 2D interaction diagrams at 600 dpi or higher.

#### 4.8.2. Drug-likeness and ADME/Tox Workflow

Five oil constituents, cinnamaldehyde, thymol, eucalyptol, p-cymene, and linoleic acid, selected from GC–MS; SMILES/SDF were taken from PubChem [46]. SwissADME computed MW, HBD/HBA, TPSA, RB, logP, and MR, and evaluated Lipinski, Veber, and Ghose rules [43,44,45,64]. pkCSM and ADMETlab 2.0 predicted HIA, logBB, logPS, CYP liabilities, clearance, and toxicity; overlapping endpoints cross-validated, single-source metrics retained [48,49,50]. Inputs used neutral microspecies at pH 7.0 and PubChem SMILES; nomenclature was harmonized with DrugBank [45,46,47]. Pass/fail flags and numerics supported reproducibility [43,44,45,48,50].

## 5. Conclusions

The simplex–centroid mixture design was used to optimize the antibacterial and antioxidant activities of thyme, cinnamon, and black seed oils, revealing synergistic effects that enhanced activity compared to the individual oils. In silico analysis identified potential molecular mechanisms and indicated generally favorable ADMET and pharmacokinetic profiles for the main constituents. Cytotoxicity testing on normal WI-38 cells showed minimal toxicity at the tested concentrations. These results indicate that the optimized oil combinations may have potential as natural agents for therapeutic and food preservation applications, while further studies are needed to confirm their safety and efficacy.

## Figures and Tables

**Table 1:** Major chemical constituents identified in thyme, cinnamon, and black seed oils by GC–MS analysis.

Oil	Compound	Chemical Class	RT (min)	RI (Exp.)	Area (%)
Thyme (*Thymus vulgaris*)	β-Pinene	Monoterpene Hydrocarbon (Bicyclic)	2.871	988	0.39
	1-Terpineol	Monoterpene Alcohol (Monocyclic)	3.142	1135	10.18
	*p*-Cymene	Aromatic Monoterpene Hydrocarbon	3.264	1021	18.69
	Eucalyptol	Monoterpene Ether	3.309	1024	20.48
	γ-Terpinene	Monoterpene Hydrocarbon (Monocyclic)	3.676	1053	1.17
	α-Terpinene	Monoterpene Hydrocarbon (Monocyclic)	4.056	1016	4.2
	Linalool	Monoterpene Alcohol (Acyclic)	4.281	1099	1.14
	Terpinen-4-ol	Monoterpene Alcohol (Monocyclic)	4.687	1175	0.54
	Borneol	Monoterpene Alcohol (Bicyclic)	5.073	1157	2.14
	α-Terpineol	Monoterpene Alcohol (Monocyclic)	5.465	1186	5.53
	Estragole	Phenylpropanoid/Aromatic Ether	5.568	1194	11.12
	Carvacrol methyl ester	Aromatic Ester	6.051	1215	0.41
	Thymol	Monoterpenoid Phenol	6.914	1291	23.11
	Caryophyllene	Sesquiterpene Hydrocarbon	8.278	1422	0.86
Cinnamon (*Cinnamomum verum*)	Benzaldehyde	Aromatic Aldehyde	2.511	957	0.59
	Benzyl alcohol	Aromatic Alcohol	2.541	1029	7.97
	Cinnamaldehyde, (E)	Phenylpropanoid/α,β-Unsaturated Aldehyde	6.907	1272	89.82
	trans-Cinnamic acid	Phenylpropanoid/α,β-Unsaturated Carboxylic Acid	8.986	1461	0.16
	14-Hydroxycaryophyllene	Sesquiterpenoid (Alcohol)	13.215	1829	0.16
	Benzyl cinnamate	Ester (Phenylpropanoid Ester)	15.352	2052	0.47
Black seed (*Nigella sativa*)	α-Thujene	Monoterpene Hydrocarbon (Bicyclic)	2.256	933	1.22
	*p*-Cymene	Monoterpene Aromatic Hydrocarbon	3.413	1020	6.73
	Thymoquinone	Benzoquinone (Monoterpenoid Quinone)	6.447	1258	4.47
	n-Hexadecanoic acid	Saturated Fatty Acid	14.685	1961	12.41
	9,12-Octadecadienoic acid (Z,Z)-	Polyunsaturated Fatty Acid	16.356	2159	74.38

**Table 2:** Matrix of the simplex–centroid design and corresponding antibacterial and antioxidant activities results.

Run	Thyme	Cinnamon	Black Seed	MIC (*E. coli*)	MIC (*S. aureus*)	DPPH_IC_50__
1	0.0	1.0	0.0	3.125	1.56	24.51 ± 2.09
2	0.333	0.333	0.333	0.781	0.781	6.27 ± 0.80
3	0.333	0.333	0.333	0.781	0.781	5.64 ± 0.48
4	0.0	0.0	1.0	6.25	6.25	23.90 ± 1.79
5	0.5	0.0	0.5	3.125	3.125	12.05 ± 0.75
6	0.5	0.5	0.0	0.781	0.781	8.92 ± 1.25
7	0.667	0.167	0.167	0.781	0.781	6.70 ± 1.18
8	1.0	0.0	0.0	3.125	1.56	13.25 ± 0.79
9	0.0	0.5	0.5	0.781	1.56	27.54 ± 1.86
10	0.0	1.0	0.0	3.125	1.56	21.85 ± 1.20
11	0.5	0.0	0.5	3.125	3.125	11.97 ± 0.83
12	0.167	0.667	0.167	0.781	0.781	15.62 ± 1.27
13	0.0	0.5	0.5	0.781	1.56	28.50 ± 1.60
14	0.5	0.5	0.0	0.781	0.781	9.85 ± 0.52
15	0.0	0.0	1.0	6.25	6.25	25.45 ± 1.73
16	0.167	0.167	0.667	3.125	3.125	13.72 ± 1.03
17	0.167	0.667	0.167	0.781	0.781	14.36 ± 1.20
18	0.167	0.167	0.667	3.125	3.125	14.88 ± 1.30
19	1.0	0.0	0.0	3.125	1.56	11.84 ± 1.91
20	0.667	0.167	0.167	0.781	0.781	7.45 ± 0.33

**Table 3:** ANOVA results for the Special Cubic model describing the effects of Thyme (A), Cinnamon (B), and Black Seed (C) oils on MIC against *E. coli* and *S. aureus*, and antioxidant activity (DPPH IC_50_).

Response	Source	df	SS	MS	F-Value	*p*-Value
*E. coli*	Model	6	58.29	9.71	89.43	<0.0001
	Residual	13	1.41	0.1086		
	Lack of fit	3	1.41	0.4707		
	Pure error	10	0.0000	0.0000		
R^2^ = 0.9763, Adj R^2^ = 0.9654, Pred. R^2^ = 0.9589						
*S. aureus*	Model	6	53.92	8.99	386.00	<0.0001
	Residual	13	0.3026	0.0233		
	Lack of fit	3	0.3026	0.1009		
	Pure error	10	0.0000	0.0000		
R^2^ = 0.9944, Adj R^2^ = 0.9918, Pred. R^2^ = 0.9903						
IC_50_ (DPPH)	Model	6	1034.25	172.37	134.20	<0.0001
	Residual	13	16.70	1.28		
	Lack of fit	3	8.12	2.71	3.16	0.0730
	Pure error	10	8.58	0.8576		
R^2^ = 0.9841, Adj R^2^ = 0.9768, Pred. R^2^ = 0.9618						

df = degree of freedom; SS = Sum of Square; MS = Mean square; R^2^ = coefficient of determination; Adj R^2^ = Adjusted; Pred. R^2^ = Predicted R^2^.

**Table 4:** Predicted and Observed Responses for the Optimal Oil Mixture.

Response	Predicted Value	Experimental Value
MIC (*E. coli*) μL/mL	0.462	0.5 ± 0.00
MIC (*S. aureus*) μL/mL	0.465	0.517 ± 0.03
DPPH IC_50_ (mg/mL)	5.56	5.32 ± 0.52

**Table 5:** ΣFIC values of binary, ternary, and optimal oil formulation against *E. coli* and *S. aureus* determined by the checkerboard assay.

Mixture Type & Composition (T:C:B)	MIC Combo *E. coli*	FIC Thyme	FIC Cinnamon	FIC Black Seed	ΣFIC *E. coli*	Interaction *E. coli*	MIC Combo *S. aureus*	FIC Thyme	FIC Cinnamon	FIC Black Seed	ΣFIC *S. aureus*	Interaction *S. aureus*
Binary (0.5:0.5:0)	0.781	0.125	0.125	0.0	0.25	Synergistic	0.781	0.25	0.25	0.0	0.50	Synergistic
Tertiary (0.167:0.667:0.167)	0.781	0.042	0.167	0.021	0.23	Synergistic	0.781	0.083	0.334	0.021	0.44	Synergistic
Binary (0:0.5:0.5)	0.781	0.0	0.125	0.063	0.19	Synergistic	1.56	0.0	0.5	0.125	0.63	Additive
Tertiary (0.1667:0.1667:0.6667)	3.125	0.167	0.167	0.335	0.67	Additive	3.125	0.334	0.334	0.334	1.0	Additive
Tertiary (0.3333:0.3333:0.3333)	0.781	0.082	0.082	0.041	0.21	Synergistic	0.781	0.167	0.167	0.042	0.38	Synergistic
Tertiary (0.6667:0.1667:0.1667)	0.781	0.167	0.042	0.021	0.23	Synergistic	0.781	0.334	0.083	0.021	0.44	Synergistic
Binary (0.5:0:0.5)	3.125	0.5	0.0	0.25	0.75	Additive	3.125	1.0	0.0	0.25	1.25	Indifferent
Optimal formulation (0.417:0.417:0.167)	0.5	0.067	0.067	0.013	0.15	Synergistic	0.517	0.139	0.139	0.014	0.29	Synergistic

**Table 6:** Predicted physicochemical properties of five major constituents derived from thyme and cinnamon essential oils and black seed oil.

Molecule	MW (g/mol)	MR Index	LogP	HBA	HBD	Lipinski’s Five Rules
Rule	≤500 (g/mol)	130 ≥ MR index ≥ 40	<5	≤10	<5	(No/Yes)
Linoleic acid	280.45	90.5	7.0	1	1	No
Cinnamaldehyde	132.16	44.6	2.2	1	0	Yes
Thymol	150.22	48.0	3.3	1	1	Yes
Eucalyptol	154.25	45.53	2.74	1	0	Yes
*p*-Cymene	134.22	45.27	3.12	0	0	Yes

**Table 7:** Prediction of ADME and Toxicity pharmacokinetic properties of five major compounds extracted from three oils (Thyme EO, Cinnamon EO, and Black seed FO).

Molecule	Absorption	Distribution		Metabolism (Substrate)		Metabolism (Inhibitor)					Excretion	Toxicity		
Compound	Human intestinal absorption (% absorbed)	Blood–brain barrier permeability (Log BB)	CNS permeability (Log PS)	CYP2D6	CYP3A4	CYP1A2	CYP2C19	CYP2C9	CYP2D6	CYP3A4	Total clearance (log ml/min/kg)	AMES test (No/Yes)	Hepatotoxicity (No/Yes)	Skin sensitization (No/Yes)
M1	90.2	0.1	−2.6	No	Yes	No	No	Yes	No	No	0.35	No	No	No
M2	95.3	0.3	−2.3	No	No	No	No	No	No	No	0.85	No	No	Yes
M3	95.00	0.50	−1.40	No	No	No	No	No	No	No	0.30	No	No	No
M4	95.52	0.541	−1.348	No	No	No	No	No	No	No	0.239	No	No	Yes
M5	95.9	0.4	−2.12	No	No	No	No	No	No	No	0.71	No	No	No

M1: Linoleic acid, M2: Cinnamaldehyde, M3: Eucalyptol, M4: p-Cymene, M5: Thymol, CNS: Central nervous system, CYP: cytochromes.

## Data Availability

The original contributions presented in this study are included in the article/Supplementary Materials. Further inquiries can be directed to the corresponding author.

## Supplementary Materials

The following supporting information can be downloaded at: https://www.mdpi.com/article/10.3390/ph19030372/s1. Figure S1: GC-MS chromatogram of thyme oil; Figure S2. GC-MS chromatogram of cinnamon oil; Figure S3. GC-MS chromatogram of black seed oil; Figure S4. Straight-line curves depict the experimental values against the expected values for each response; Table S1: ANOVA for Special Cubic model (Response 1: MIC E. coli); Table S2: ANOVA for the Special Cubic model (Response 2: MIC S. aureus); Table S3: ANOVA for Special Cubic model (Response 3: DPPH IC50).
