# Supplementary material for: Optimization of Thyme, Cinnamon, and Black Seed Oil Combinations for Enhanced Antibacterial and Antioxidant Efficacy: Mixture Design and In Silico Insights
Source: Pharmaceuticals (Basel). 2026 Feb 26;19(3):372. doi: 10.3390/ph19030372 (PMC13029202; doi:10.3390/ph19030372)
Supplement: Supplementary file 1 [file pharmaceuticals-19-00372-s001.zip › pharmaceuticals-4095298-supplementary.pdf]

Optimization of Thyme, Cinnamon, and Black Seed Oil Combinations for Enhanced Antibacterial and Antioxidant Efficacy :Mixture-Design and In Silico Insights

Mahmoud S. Maher[1], Dina A. Altwiley[2], Dalal M. Alkuraythi[2], Mahmoud M. A. Moustafa[3], Mary S. Khalil[1], Tarek A. A. Moussa[1], Nawal Magdy [1]

[1]Botany and Microbiology Department, Faculty of Science, Cairo University, Giza 12613, Egypt

[2]Department of Biological Sciences, University of Jeddah, Jeddah, Saudi Arabia

[3]Genetics and Genetic Engineering Dept., Faculty of Agriculture, Benha University, Egypt.

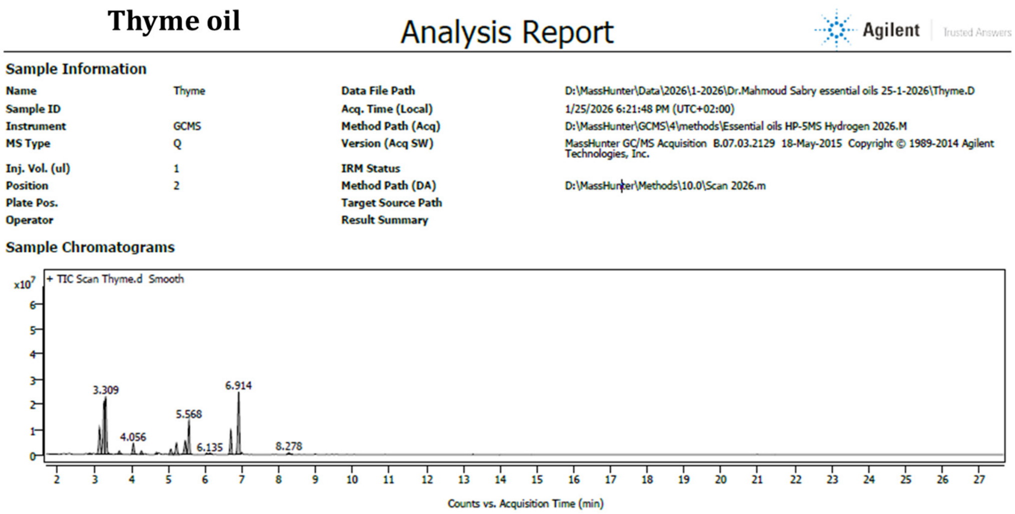

Figure S1. GC-MS chromatogram of thyme oil

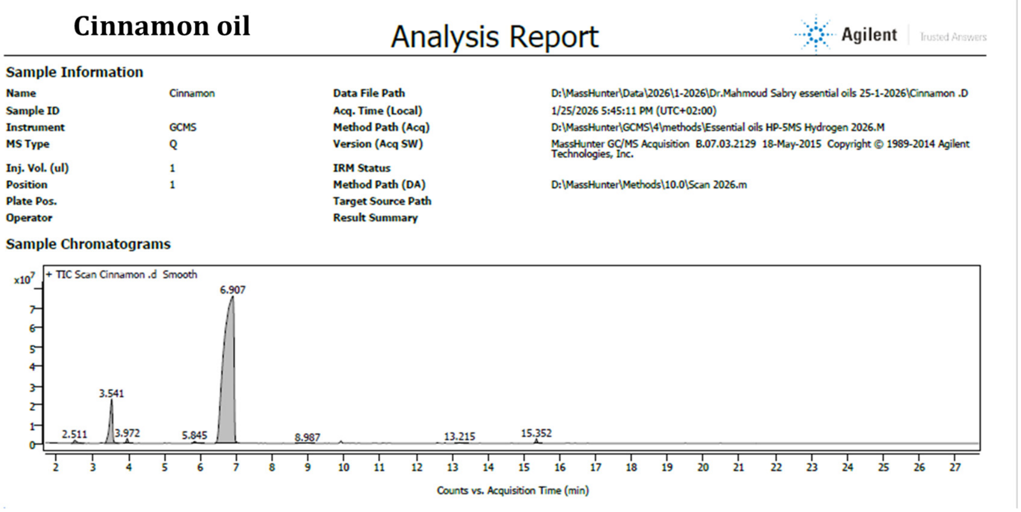

Figure S2. GC-MS chromatogram of cinnamon oil

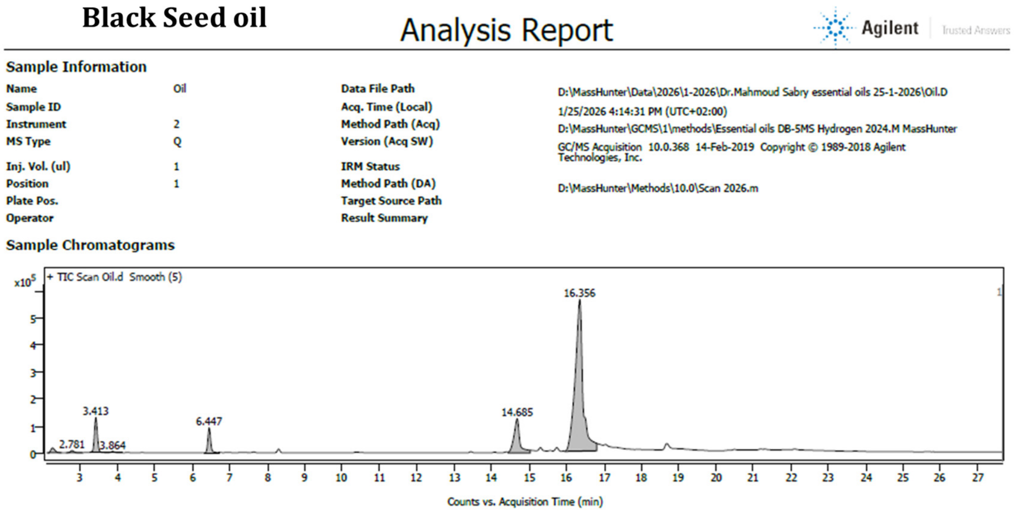

Figure S3. GC-MS chromatogram of black seed oil

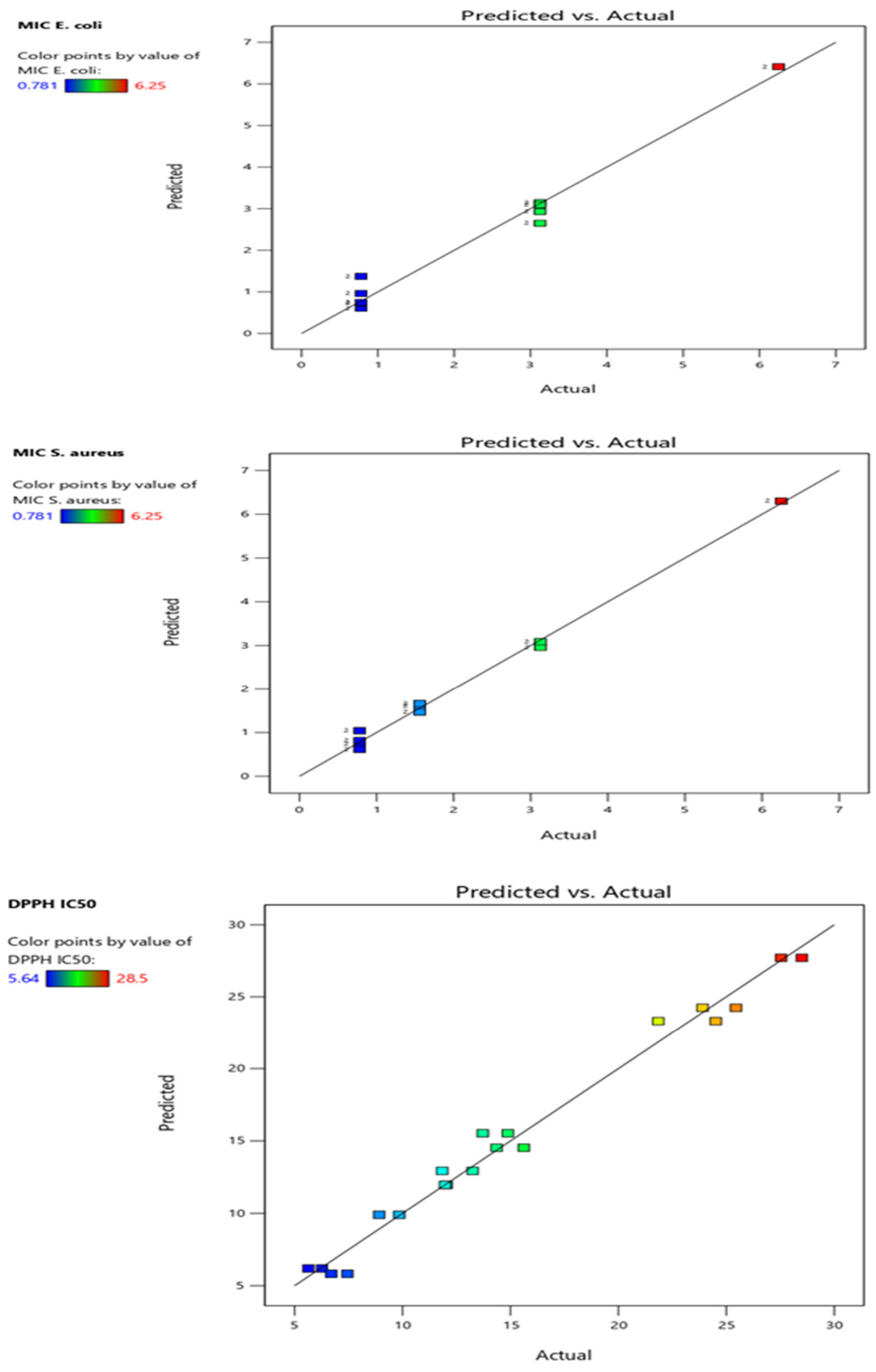

Figure S4. Straight-line curves depict the experimental values against the expected values for each response.

Table S1

ANOVA for Special Cubic model

Response 1: MIC E. coli

Source	Sum of Squares	df	Mean Square	F-value	p-value	
**Model**	58.29	6	9.71	89.43	< 0.0001	significant
(1)Linear Mixture	22.11	2	11.06	101.77	< 0.0001	
AB	7.98	1	7.98	73.50	< 0.0001	
AC	3.37	1	3.37	30.98	< 0.0001	
BC	19.67	1	19.67	181.12	< 0.0001	
ABC	0.0053	1	0.0053	0.0489	0.8284	
**Residual**	1.41	13	0.1086			
Lack of Fit	1.41	3	0.4707			
Pure Error	0.0000	10	0.0000			
**Cor Total**	59.70	19				

Table S2

ANOVA for the Special Cubic model

Response 2: MIC S. aureus

Source	Sum of Squares	df	Mean Square	F-value	p-value	
**Model**	53.92	6	8.99	386.00	< 0.0001	significant
(1)Linear Mixture	39.63	2	19.82	851.21	< 0.0001	
AB	0.8622	1	0.8622	37.04	< 0.0001	
AC	0.8646	1	0.8646	37.14	< 0.0001	
BC	7.11	1	7.11	305.42	< 0.0001	
ABC	0.4788	1	0.4788	20.57	0.0006	
**Residual**	0.3026	13	0.0233			
Lack of Fit	0.3026	3	0.1009			
Pure Error	0.0000	10	0.0000			
**Cor Total**	54.22	19				

Table S3

ANOVA for Special Cubic model

Response 3: DPPH IC50 (1)

**Source**	**Sum of Squares**	**df**	**Mean Square**	**F-value**	**p-value**	
**Model**	1034.25	6	172.37	134.20	< 0.0001	significant
[(1)]Linear Mixture	508.76	2	254.38	198.05	< 0.0001	
AB	91.30	1	91.30	71.09	< 0.0001	
AC	59.29	1	59.29	46.16	< 0.0001	
BC	20.95	1	20.95	16.31	0.0014	
ABC	117.85	1	117.85	91.75	< 0.0001	
**Residual**	16.70	13	1.28			
Lack of Fit	8.12	3	2.71	3.16	0.0730	not significant
Pure Error	8.58	10	0.8576			
**Cor Total**	1050.95	19				
